# DNA Methylation and Regulation of the *CD8A* after Duck Hepatitis Virus Type 1 Infection

**DOI:** 10.1371/journal.pone.0088023

**Published:** 2014-02-04

**Authors:** Qi Xu, Yang Chen, Wen Ming Zhao, Zheng Yang Huang, Yang Zhang, Xiu Li, Yi Yu Tong, Guo Bing Chang, Xiu Jun Duan, Guo Hong Chen

**Affiliations:** 1 Key Laboratory of Animal Genetics & Breeding and Molecular Design of Jiangsu Province, Yangzhou University, Yangzhou, People's Republic of China; 2 National Waterfowl Germplasm Resource Pool, Taizhou, People's Republic of China; The University of Melbourne, United States of America

## Abstract

**Background:**

Cluster of differentiation 8 (CD8) is expressed in cytotoxic T cells, where it functions as a co-receptor for the T-cell receptor by binding to major histocompatibility complex class I (MHCI) proteins, which present peptides on the cell surface. *CD8A* is critical for cell-mediated immune defense and T-cell development. *CD8A* transcription is controlled by several cis-acting elements and trans-acting elements and is also regulated by DNA methylation. However, the epigenetic regulation of *CD8A* in the duck and its relationship with virus infection are still unclear.

**Results:**

We investigated the epigenetic transcriptional regulatory mechanisms, such as DNA methylation, for the expression of the *CD8A* and further evaluated the contribution of such epigenetic regulatory mechanisms to DHV-I infection in the duck. Real-time quantitative polymerase chain reaction (RT-qPCR) revealed the highest level of *CD8A* expression to be in the thymus, followed by the lungs, spleen, and liver, and the levels of *CD8A* expression were very low in the kidney, cerebrum, cerebellum, and muscle in the duck. RT-qPCR also demonstrated that the *CD8A* mRNA was down-regulated significantly in morbid ducklings treated with DHV-1 and up-regulated significantly in non-morbid ducklings in all the tissues tested. In addition, hypermethylation of *CD8A* was detected in the morbid ducklings, whereas relatively low methylation of *CD8A* was evident in the non-morbid ducklings. The *CD8A* mRNA level was negatively associated with the CpG methylation level of *CD8A* and global methylation status.

**Conclusions:**

We concluded that the mRNA level of the *CD8A* was negatively associated with the CpG methylation level of *CD8A* and global methylation status in the duck, suggesting that the hypermethylation of CD8A may be associated with DHV-1 infection. The first two CpG sites of the *CD8A* promoter region could be considered as epigenetic biomarkers for resistance breeding against duckling hepatitis disease in the duck.

## Introduction

The CD8 molecule is composed of two chains that are encoded by two distantly related but closely linked genes, *CD8A* and *CD8B* [1]. Because CD8B lacks the cysteine motif critical to lymphocyte cell kinase (Lck) binding, the functions of the CD8 molecule are concentrated on the α chain [2]. CD8A is critical for cell-mediated immune defense and T-cell development [3]. In mice, *CD8A* transcription is controlled by several cis-acting elements, including enhancers E8 I∼E8 V and trans-acting elements such as GATA-3 and CREB [4]–[8]. In addition, *CD8A* expression was shown to be regulated by DNA methylation in humans [9]–[11]. Thus, the epigenetic regulation of *CD8A* is very important.

Duck hepatitis is a highly fatal, rapidly spreading viral infection of young ducklings, characterized primarily by hepatitis. It is mainly caused by duck hepatitis virus type 1 (DHV-1) [12]. It is the most widely distributed virus that can cause mortality rates above 90% in 1-week-old ducklings [13]. During the course of DHV-1 infection, TNFA, IL6, CD3, CD4 and CD8A gene expression are increased in humans. The relationship between promoter methylation of some genes (e.g., methylation of p16 RAS-related structure domain family gene 1, glutathione S transferase 1, and E-cadherin) and hepatitis has caused increased concern [14]–[17]. Thus, more extensive investigations are needed to understand the detailed processes of epigenetic regulation. Ducks are considered as a feasible model of animal infection for the hepatitis B virus, which is the causative agent of acute and chronic hepatitis B in humans [14]. However, the epigenetic regulation of *CD8A* in the duck and its relationship with virus infection remain unclear.

In the present study, promoter methylation, transcription of the *CD8A*, and the global DNA methylation level after DHV-1 infection, as well as the relationship between these factors and regulation of expression, were analyzed. This information might facilitate better understanding of the expression control of *CD8A* by ascertaining the epigenetic status in the *CD8A* promoter and *CD8A* expression in relation to DHV-1 infection.

## Materials and Methods

### Ethics statement

All animal experiments were reviewed and approved by the Institutional Animal Care and Use Committee of Yangzhou University. Experiments were performed in accordance with the Regulations for the Administration of Affairs Concerning Experimental Animals of Yangzhou University (Yangzhou University, China, 2012) and the Standards for the Administration of Experimental Practices (Jiangsu, China, 2008). All operations were performed according to recommendations proposed by the European Commission (1997), and all efforts were made to minimize suffering.

### Ducklings, viral infections, and sample collection

Because separating pure primary duck hepatocytes is very difficult, we carried out the experiments in animals. During the pilot study, the 30 3-day-old ducklings were obtained from the Chinese Waterfowl Germplasm Resource Pool (Taizhou, China). The ducklings were determined to be free of DHV-1 by amplification of conserved regions of the DHV-1 3D gene using reverse transcription-polymerase chain reaction (RT-PCR) [18] with the primers shown in Table 1. To determine the proper challenge dose, the ducklings were intramuscularly inoculated with different doses of allantoic liquid containing DHV-1. Euthanasia was not employed, and death was used as the endpoint so that symptom changes and mortality could be accurately recorded. Some infected ducklings appeared depressed and had little desire for food 24 h.p.i. (hours post infection), and 30% (9/30) of the infected birds died of typical hepatitis when the ducklings were inoculated with 0.4 mL of normal saline. The death peak was approximately 36 h.p.i., and the mortality rate declined dramatically by 48 h.p.i. After 72 h.p.i., no duck deaths were observed, which is in agreement with data previously described in the literature [12]–[13].

**Table 1:** Oligonucleotide primers used in the experiments.

Primer name	Primer sequence (5′→3′)	Annealing temperature (°C)	Usage
DHV-13DF	ACAATGACCCAGCCTTAG	56	DHV-13D gene amplification
DHV-13DR	CCACTGTATCTTCCCTTC		
e*CD8A*F	GAAGTCCTTCAAGGCAGAG	60	RT-qPCR
e*CD8A*R	AGACGTCCCTCTTGGTGAC		
GAPDH-F	TGCTAAGCGTGTCATCATCT	60	RT-qPCR
GAPDH-R	AGTGGTCATAAGACCCTCCA		
M*CD8A*F1	TTGTGATTTTGAATTTTTAGTGAAG	60	Methylation detection
M*CD8A*R1	ATAACCAAAAATCCCAATCCC		
M*CD8A*F2	TTTGGAATAGAAATGAATTTTTTTAGAA	60	Methylation detection
M*CD8A*R2	ATACTCACAAAACCCCAAACTAAAC		

The 80 3-day-old ducklings used in the main study were intramuscularly inoculated with DHV-1 successfully. The ducklings were randomly divided into two groups. Forty ducklings were inoculated with 0.4 mL of allantoic liquid containing DHV-1 (based on the results of pilot study), and 40 received normal saline (as uninfected controls). The two groups were maintained in separate rooms. The infected ducklings were monitored every 30 mins. Once some of them (morbid ducklings) displayed typical symptoms of DHV-1 infection, they were immediately anesthetized with sodium pentobarbital (intraperitoneal injection; 150 mg/kg) and killed by exsanguination. The heparinized blood and tissue samples (thymus, lung, spleen, liver, kidney, cerebrum, cerebellum, and muscle) were obtained promptly. The tissues were snap-frozen in liquid nitrogen immediately and stored at −80°C until needed. The other ducklings (non-morbid ducklings) were sampled after 7 days, and the uninfected ducklings were sampled after 7 days.

### Real-time quantitative PCR (RT-qPCR) analysis of CD8A expression in the duck

Total RNA extracted from each tissue was subjected to RT-qPCR analysis. Single-strand cDNA was synthesized using approximately 5 mg of total RNA and a Revertaid™ First Strand cDNA Synthesis kit (Fermentas, Fermentas China Co., Ltd, China), and the cDNA was diluted 10 times. Diethylpyrocarbonate-treated water was used as the negative control, and the primers used are shown in Table 1. Template without reverse transcriptase was used to check for contaminating genomic DNA. A SYBR Green RT-qPCR assay was conducted to determine the pattern of *CD8A* expression, and the primers are shown in Table 1. The PCR temperature profile and reaction conditions were performed as specified by the manufacturer's instructions for SYBR Premix Ex Taq™ (TaKaRa, Takara Biotechnology (Dalian) Co., Ltd, China) in an ABI two-step RT-qPCR system (Applied Biosystems 7500, U.S.). The relative expression was calculated using the 2^−ΔΔCt^ method [19]. The *GAPDH* served as an internal reference gene, and muscle from healthy ducks served as a calibrator tissue for analysis of differential expression. For analysis of expression patterns in infected ducks, the mean ΔCt value of the control duck within each group was used as the calibrator.

### Bisulfite genomic sequencing of the CD8A promoter

Genomic DNA of all peripheral blood samples was extracted from the tissues using the Quick-gDNA™ MiniPrep Kit (Zymo Research, Zymo Research Corp., USA) according to the manufacturer's instructions. Bisulfite treatment of the genomic DNA was performed using the DNA Modification Kit (Zymo Research, Zymo Research Corp., USA). The CpG island of the *CD8A* promoter and exon 1 (GenBank accession number: JX051841 and JX051831) was predicted using online software (http://www.urogene.org/methprimer/index1.html), and the primers were designed for the *CD8A* promoter and exon 1 using MethPrimer, a program for designing bisulfite-conversion-based methylation PCR primers. The modified DNA was amplified by PCR using the two sets of primers shown in Table 1. These primers were specific for the converted DNA, but did not contain any CpG sites in their sequence; therefore, both methylated and unmethylated DNA could be amplified by the same primer sets. Each PCR mixture contained genomic DNA. The PCR products were sequenced on an ABI automated sequencer 3100 (ABI, USA).

### Determination of global DNA methylation

Global DNA methylation was determined using the MethylFlash Methylated DNA Quantification Kit (Epigentek, Epigentek Group Inc., USA). The kit measures the methylcytosine content as a percentage of the total cytosine content. Using a DNA concentration of 20 µg/mL, the purified DNA was added to the enzyme-linked immunosorbent assay (ELISA) plate. The methylated fraction of DNA is quantified using 5-methylcytosine-specific antibodies. The amount of methylated DNA was proportional to the optical density (OD) intensity in an ELISA plate reader at 450 nm. DNA methylation was calculated using the following formula [20]:
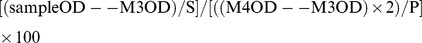
where OD is the optical density, M3 is the negative control, an unmethylated polynucleotide containing 50% cytosine, S is the amount of input sample DNA in ng, M4 is the positive control, a methylated polynucleotide containing 50% 5-methylcystosine, and P is the amount of input positive control in ng. The amount of methylated DNA was expressed as a percentage of the total DNA.

### Statistical analysis

Statistical analyses were conducted using the SPSS 13.0 package. Point-wise comparison was performed to analyze the difference in methylation content at different CpG sites. A *t*-test was performed to analyze expression levels of *CD8A*. We also performed regression analysis between the DNA methylation content and mRNA level of *CD8A*.

## Results

### Clinical symptoms and DHV-13D gene detection after DHV-1 infection

**Figure 1:**
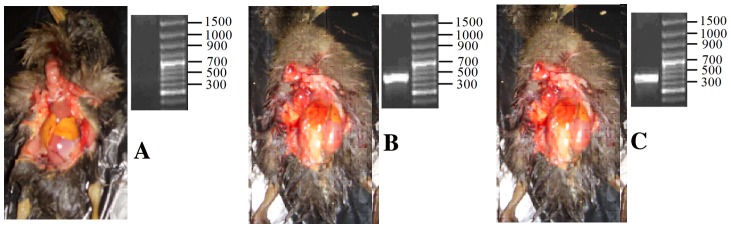
Duck model establishment and verification of infection with DHV-1. A: No symptoms were evident in duckling organs without the specific bands of the conserved regions in the *DHV-13D* gene in the control group. B and C: Some symptoms were observed in duckling organs, including an enlarged liver with yellow or yellow-brown spots on kidneys, hyperemia and swelling, spleen enlargement with the specific bands of the conservative regions in the *DHV-13D* gene in the experimental ducklings (B: morbid group; C non-morbid group).

Necropsy revealed an enlarged liver with yellow or yellow-brown spots on kidneys, hyperemia and swelling, and spleen enlargement in about 30% (11/40) of experimental ducklings. The results are shown in Figure 1. The other infected ducklings were typically asymptomatic. To confirm whether the ducks acquired DHV-1 infection, the specific bands of the conservative regions in the *DHV-13D* gene were amplified in experimental ducklings (morbid group and non-morbid group). This outcome indicated that the infected duck hepatitis virus animal model was constructed successfully.

### Expression of CD8A mRNA in different duck tissues

**Figure 2:**
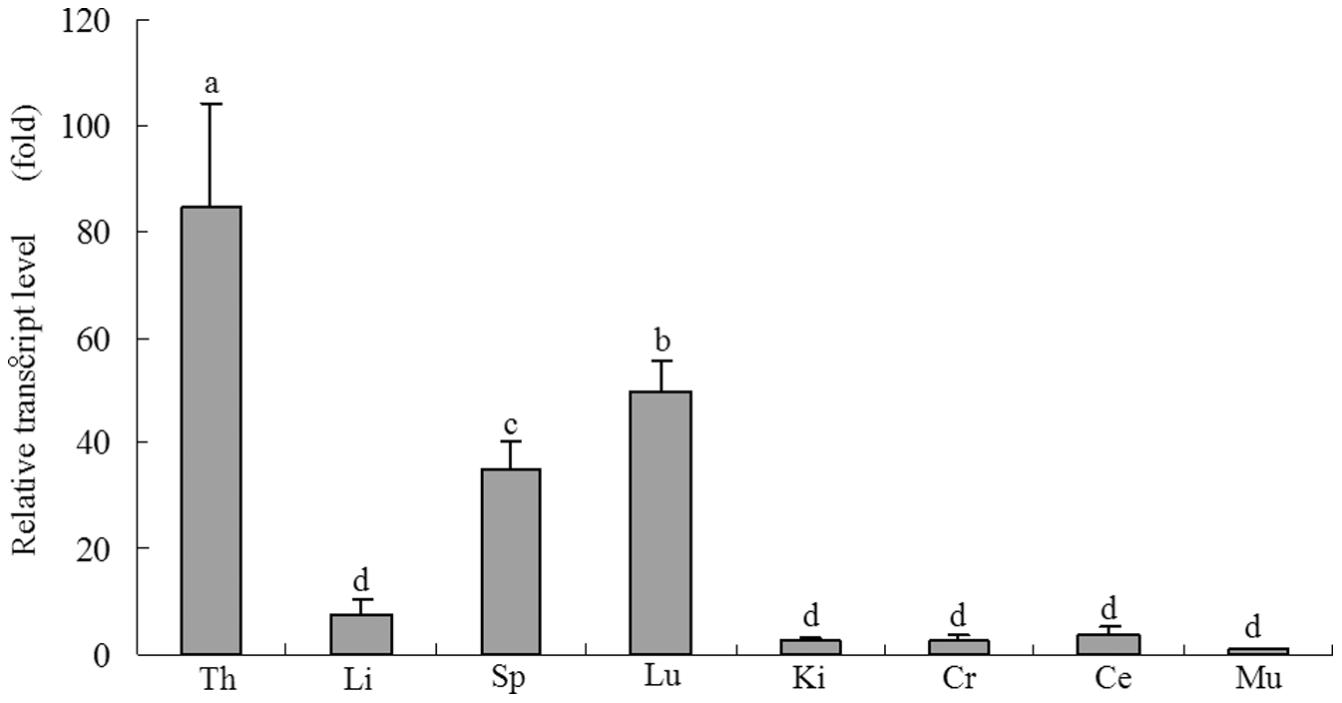
Expression profile of *CD8A* transcripts in duck tissues. Tissues analyzed include the thymus (Th), liver (Li), spleen (Sp), lung (Lu), kidney (Ki), cerebrum (Cr), cerebellum (Ce), and muscle (Mu). Expression data for each tissue were analyzed from three randomly selected individuals. Vertical bars represent the mean±standard deviation (S.D.) (n = 3). Significant differences relative to controls are indicated with adjacent letters (*P*<0.05) and with separate letters (*P*<0.01).

Real-time RT-PCR analysis of *CD8A* mRNA showed the location of the highest level of expression to be the thymus followed by the lungs, spleen, and liver (Figure 2). The mRNA levels were about 10 times greater in the thymus than in most other tissues (*p*<0.01). Little or no *CD8A* expression was observed in the kidneys, cerebrum, cerebellum, or muscle (Figure 2).

### Differential expression of CD8A mRNA after DHV-1 infection in duck

**Figure 3:**
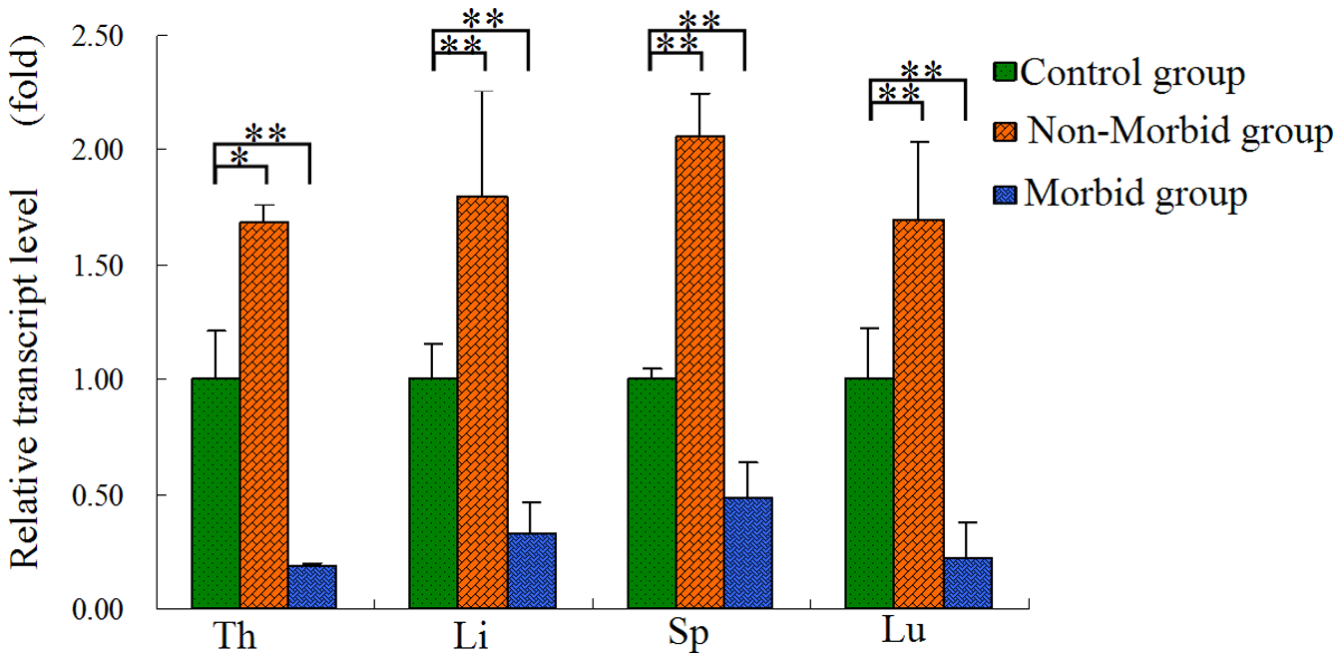
*CD8A* expression in duckling tissue treated with DHV-1. Tissues analyzed include the thymus (Th), liver (Li), spleen (Sp), lung (Lu). Expression data for each tissue were analyzed from three randomly selected individuals. Vertical bars represent the mean±S.D. (n = 3). Significant differences relative to controls were indicated with * (*P*<0.05) and ** (*P*<0.01).

To evaluate differential expression of *CD8A* mRNA after DHV-1 infection, four tissue types (i.e., thymus, liver, spleen, and lung) were chosen according to the results of previous analyses of tissue-specific expression. The transcript levels of *CD8A* were detected in morbid ducklings, non-morbid ducklings, and control ducklings (Figure 3). compared with the control ducklings, *CD8A* mRNA was down-regulated very significantly in morbid ducklings and up-regulated very significantly in non-morbid ducklings in all four tissue types (*p*<0.01), beyond that up-regulated significantly in non-morbid ducklings of thymus (*p*<0.05).

### Analysis of CD8A promoter methylation in duck

**Figure 4:**
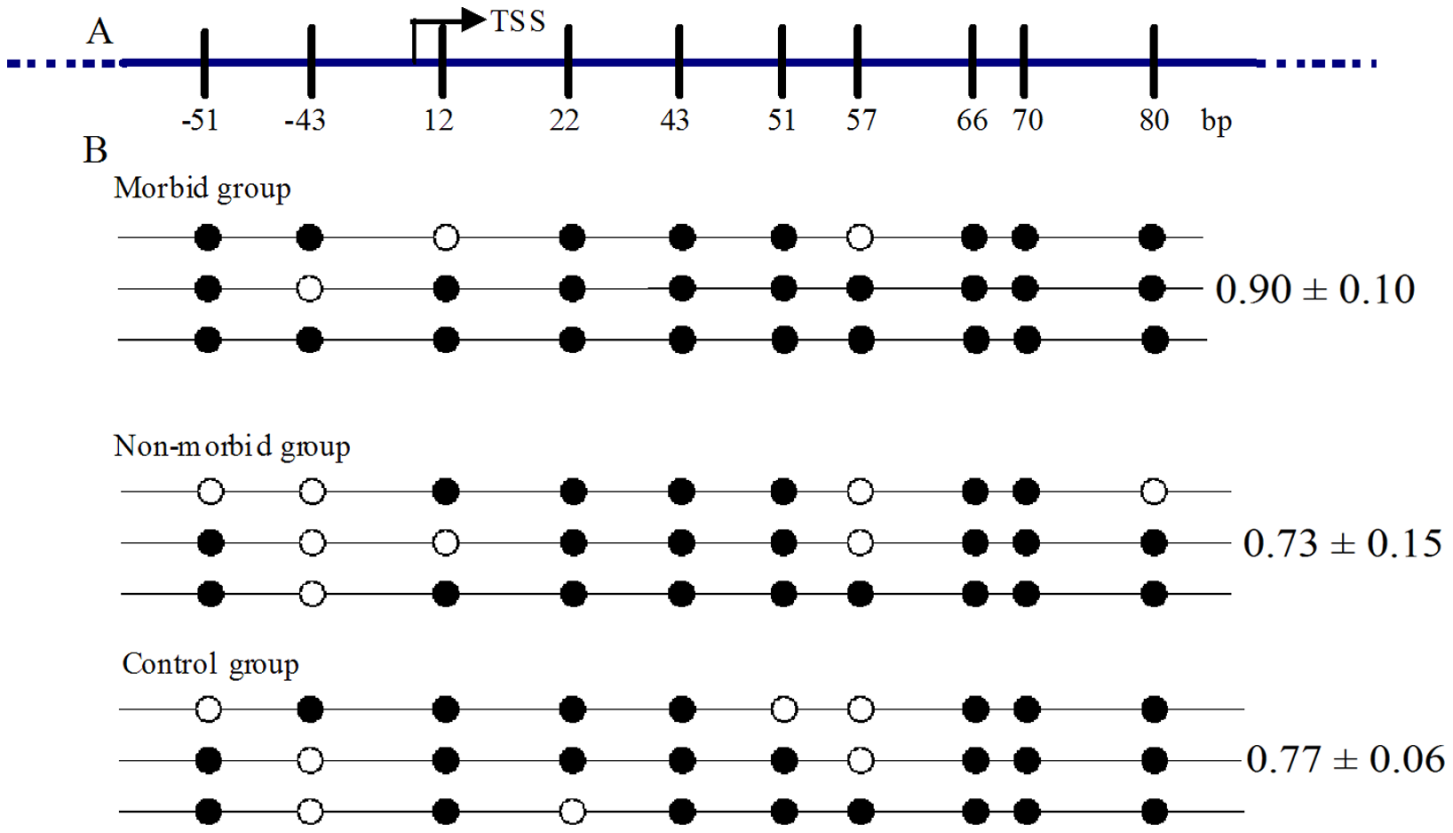
The differential methylation level of the *CD8A* promoter in the duck. (A) The schematic diagram of the *CD8A* promoter. An arrow indicates the transcription start site (TSS) (+1 bp), and short vertical lines indicate the positions of the methylation sites relative to the TSS. Ten CpG islands were detected in the region. (B) Bisulfite sequencing analysis of the DNA methylation profile of the individual CpG sites in the *CD8A* promoter in the morbid, non-morbid, and control groups. The solid and open circles indicate methylation and unmethylation status, respectively. Each PCR product was subcloned, and three samples were subjected to sequencing analysis (n = 3, mean ± S.D.).

**Figure 5:**
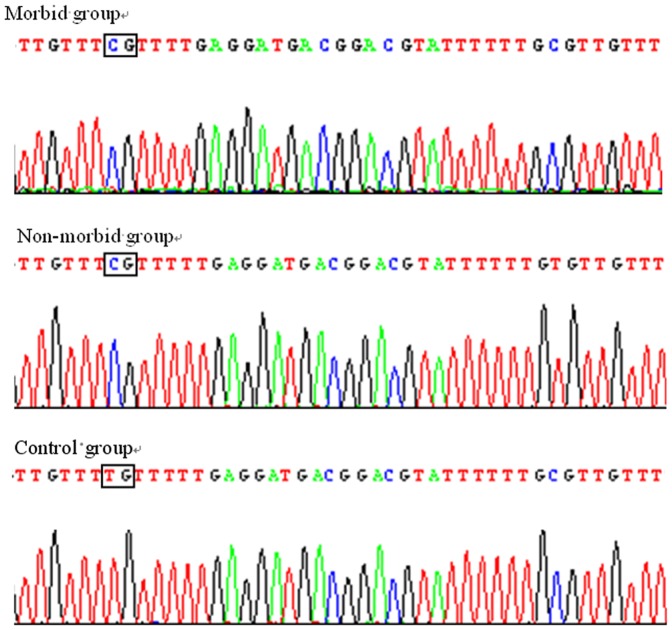
Examples of bisulfite genomic sequencing chromatography. DNA was amplified and sequenced using each primer set on an ABI automated sequencer with dye terminators. After bisulfite treatment, unmethylated cytosine was converted to thymine, while methylated cytosine was invariable in the CpG sites.

Ten CpG methylation sites were identified flanking the transcription start site (TSS) of the *CD8A* in the duck (Figure 4a). Bisulfite DNA sequencing was conducted to assess the extent of CpG island methylation in the *CD8A* promoter in the peripheral blood of morbid ducklings and non-morbid ducklings infected with DHV-1. Results are shown in Figure 4b. Examples of such sequencing are presented in Figure 5. We noted that after bisulfite treatment, unmethylated cytosine was converted to thymine. Hypermethylation of *CD8A* was detected in the morbid ducklings (0.90±0.10), whereas the relative methylation of *CD8A* was 0.73 and 0.77 in the non-morbid ducklings and control ducklings, respectively. In addition, CpG methylation upstream of the TSS (the first two CpG sites) was significantly different among the three groups, and the methylation of the *CD8A* was higher in the morbid ducklings than in the other ducklings.

### Quantitative evaluation of global methylation status

**Figure 6:**
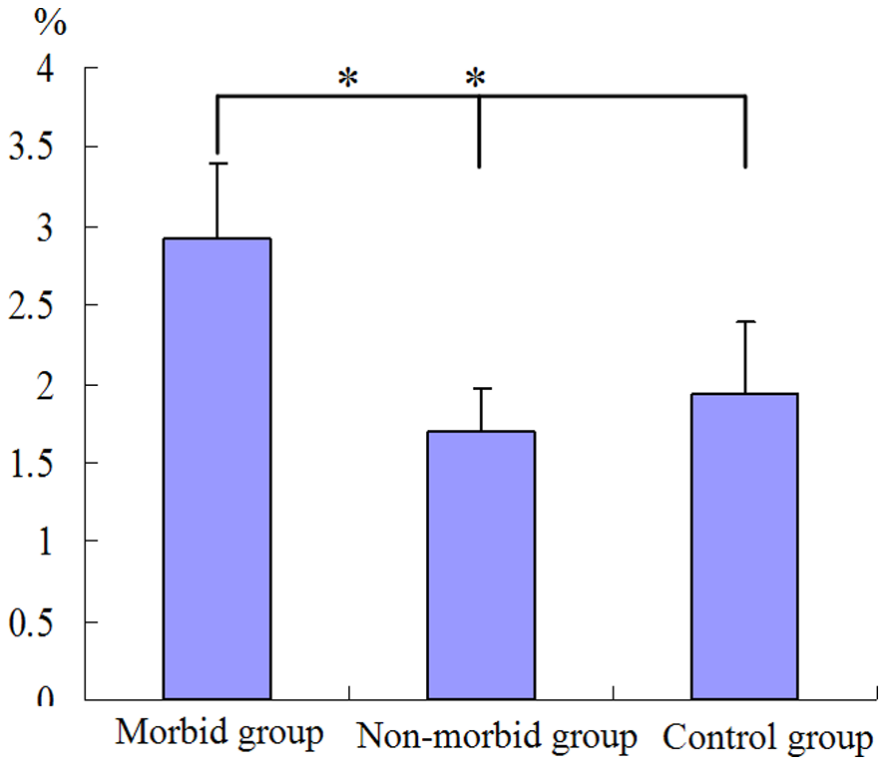
Comparison of global DNA methylation among morbid, non-morbid and control ducklings isolated from peripheral blood treated with DHV-1. Significant difference in global DNA methylation between morbid ducklings and non-morbid ducklings. * means *P*<0.05 (n = 3).

In order to determine whether associations exist between *CD8A* and global genome methylation, we employed an ELISA-based assay (Epigentek, Epigentek Group Inc., USA) to quantitatively measure genomic methylation. The global genomic DNA methylation levels were significantly higher in morbid ducklings than in non-morbid ducklings and control ducklings (*p*<0.05,Figure 6). In this sense, the global genomic methylation level was consistent with *CD8A* methylation in morbid ducklings.

### Association analysis between mRNA levels and DNA methylation content

To further clarify the association between *CD8A* mRNA and DNA methylation levels in the duck, multivariable statistics analysis was performed to explore the relationship among the DNA methylation level of *CD8A*, global methylation status, and *CD8A* mRNA level. Multiple regression equations were fitted. Taken together, we found that the higher the methylation levels, the lower the mRNA level in all tissues combined and in the lung alone (*p*<0.05 or *p*<0.01).



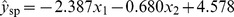


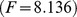
;



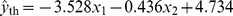


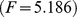
;



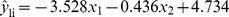


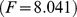
;



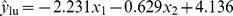


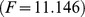


Where 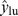
is the RNA expression level in tissue (thymus, lungs, liver or spleen), 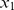 is the DNA methylation level of *CD8A*, and 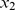 is the global methylation status.

## Discussion

In the present study, the relative expression pattern of *CD8A* mRNA was investigated in various tissues by RT-qPCR analysis, which indicated a high level of expression in immune-associated tissues (i.e., thymus, spleen, lungs, and liver); particularly in the thymus, *CD8A* mRNA was 10 times higher than the levels in all other tissue types reported to date [21]–[23]. Because the thymus is the primary lymphoid organ responsible for T-cell maturation in vertebrates, higher expression in this tissue is expected [24]. High *CD8A* mRNA in the spleen would also be expected because this organ is involved in immunological responses [25], [26]. Expression of *CD8A* in duck lungs and liver is most likely due to the presence of CD8-positive cells in those tissues. The relative level of gene expression was analyzed after infection with DHV-1. There was a significant difference in the level of *CD8A* expression between morbid ducklings and non-morbid ducklings. The *CD8A* mRNA level was up-regulated significantly in non-morbid ducklings and down-regulated significantly in morbid ducklings in all four tissue types. Similar results concerning *CD8A* mRNA in the spleen and thymus following viral and bacterial infection has been reported [27]–[29]. CD8A plays a very important role, as a T lymphocyte surface marker, in the antiviral response and the cellular immune system [30], [31]. CD8-positive cells might work quickly after DHV-1 infection to form a CD8-major histocompatibility complex (MHC) I-antigen peptide complex, which function together to target cells, resulting in target-cell apoptosis [32]. High expression of *CD8A* might help to form more CD8-MHC I-antigen peptide complexes, eventually leading to additional virus-infected target cell apoptosis in non-morbid ducklings.

We found that the DNA methylation level of the *CD8A* promoter and global methylation status were altered following DHV-1 infection. Our bisulfite genomic sequencing revealed that DNA methylation of the *CD8A* promoter could repress *CD8A* transcription in peripheral blood. It is well known that DNA methylation plays an important role in transcriptional regulation in mammals [33]–[35]. For example, the methylation fluctuation of the *CD8A* in mice was related to *CD8* enhancer-mediated recruitment of the zinc finger protein: zinc-finger protein-related factor(MAZR)[8].

In the present study, we thus found that the methylation level of the first two CpG sites of the *CD8A* significantly differed among the three groups. We predicted a transcription factor binding site and found trans-action element (E47) flanking the first two CpG sites. The two CpG sites might be related to a transcription factor of the E47 binding site. Next, we verified the methylation status of the first two CpG islands to determine whether the E47 binding site was affected and to confirm the regulation of *CD8A* expression.

In our previous study, we screened for single nucleotide polymorphisms (SNP) in the coding sequence of *CD8A* between morbid and non-morbid ducklings, but no SNPs were detected. Therefore, the first two CpG islands of the *CD8A* promoter region could be considered as an epigenetic biomarker in breeding for DHV-1 resistance. In general, methylation status fluctuation is a key factor associated with disease status and includes global genome methylation level reduction and local CpG island methylation increase, which will lead to genome instability [36]. Interestingly, we found the global genomic methylation level and specific gene (*CD8A*) methylation to be higher in morbid ducklings with DHV-1 infection due to multiple gene methylation phenomenon or a cascade methylation effect. It was reported that multiple gene methylation existed in human hepatitis disease [37]–[39]. Thereafter, we explored the relationship between the DNA methylation level and mRNA level. There was a negative effect of the methylation levels of the *CD8A* promoter region on its expression, a finding similar to that of other reports [40]–[42].

## Conclusion

In conclusion, our data defined the CpG site methylation patterns of the *CD8A* promoter regions in morbid ducklings and non-morbid ducklings infected with DHV-1. The results found that the expression levels of the *CD8A* were negatively associated with the CpG methylation level of *CD8A* and global methylation status, suggesting that the hypermethylation profiles might contribute to DHV-1 infection. The first two CpG sites of the *CD8A* promoter region may be epigenetic biomarkers for resistant breeding against duckling hepatitis disease in the duck.
