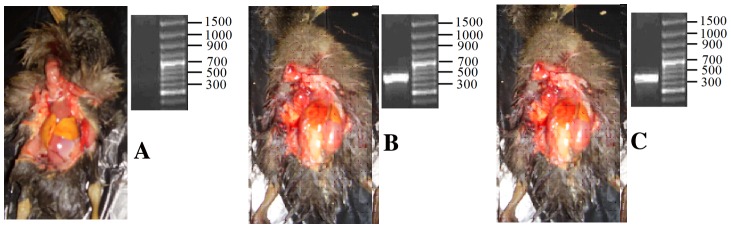# Correction: DNA Methylation and Regulation of the *CD8A* after Duck Hepatitis Virus Type 1 Infection

**DOI:** 10.1371/journal.pone.0091469

**Published:** 2014-02-28

**Authors:** The PLOS ONE Staff

The name of the eighth author is spelled incorrectly. The correct name is: Guo Bin Chang.

In addition, Figure 1 is incorrect. Please view a corrected version of Figure 1 here:

**Figure:**