# Correction: DNA Methylation and Regulation of the *CD8A* after Duck Hepatitis Virus Type 1 Infection

**DOI:** 10.1371/journal.pone.0095650

**Published:** 2014-04-14

**Authors:** The PLOS ONE Staff

There is an error in the Correction posted on February 28, 2014. Please view the correct Figure 1 here.

**Figure 1:**
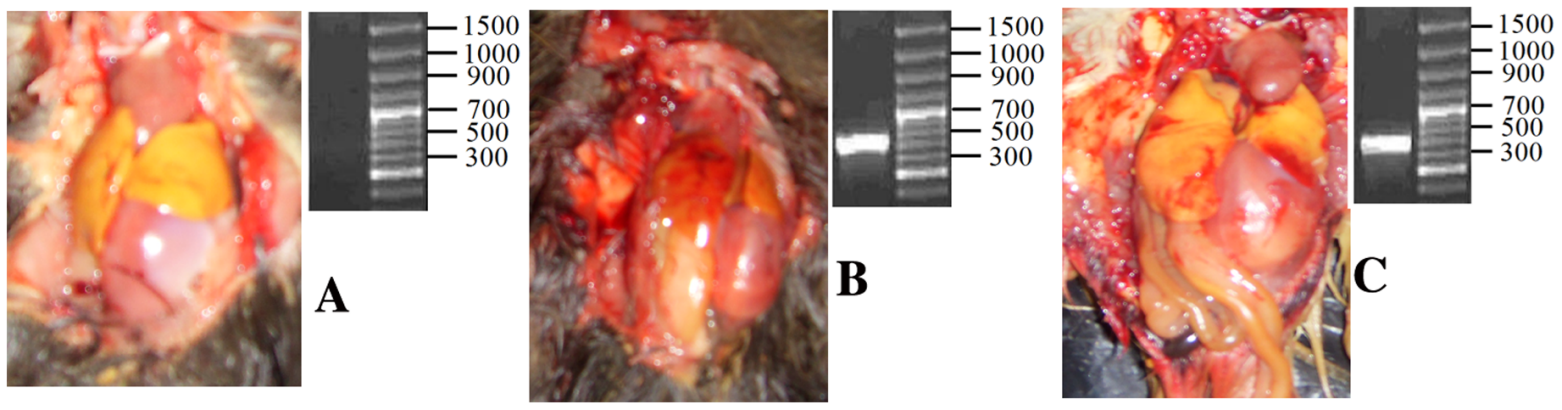
Duck model establishment and verification of infection with DHV-1. A: No symptoms were evident in duckling organs without the specific bands of the conserved regions in the *DHV-13D* gene in the control group. B and C: Some symptoms were observed in duckling organs, including an enlarged liver with yellow or yellow-brown spots on kidneys, hyperemia and swelling, spleen enlargement with the specific bands of the conservative regions in the *DHV-13D* gene in the experimental ducklings (B: morbid group; C non-morbid group).
